# Editorial: Immune-mediated disorders of the spinal cord: Diagnosis, treatment strategies, and outcomes in the 21st century

**DOI:** 10.3389/fneur.2022.1087631

**Published:** 2022-11-29

**Authors:** Giulia Fadda, Eoin P. Flanagan, Elia Sechi

**Affiliations:** ^1^Department of Neurology and Neurosurgery, McGill University, Montreal, QC, Canada; ^2^Department of Neurology, Mayo Clinic, Scottsdale, AZ, United States; ^3^Department of Laboratory Medicine and Pathology, Mayo Clinic, Rochester, MN, United States; ^4^Department of Medical, Surgical and Experimental Sciences, Faculty of Medicine and Surgery, University of Sassari, Sassari, Italy

**Keywords:** myelitis, myelopathy, MOGAD, NMOSD, multiple sclerosis, paraneoplastic, immune checkpoint inhibitors, iatrogenic myelitis

The last 20 years have seen important advances in the diagnosis and treatment of spinal cord disorders (1, 2). The growing number of specific biomarkers and MRI signs associated with different myelopathies has given a definite identity to diseases previously labeled as idiopathic (e.g., “idiopathic acute transverse myelitis”) (3). An integrated approach combining essential clinical (*e.g*., timing of symptoms presentation) and imaging (e.g., length of the T2-lesion on spinal cord MRI) characteristics is now fundamental to orient the diagnostic suspicion across different myelopathy etiologies (Figure 1) (4–6). Specific diagnoses might require different treatment approaches, and physicians are asked to navigate this evolving landscape posing the right indication for diagnostic testing and giving the correct interpretation to the laboratory results. In this context, immune-mediated disorders of the spinal cord are of particular importance given the potential for reversibility with prompt and appropriate treatment. This category of disorders include both demyelinating and non-demyelinating myelitis, and emerging disease entities complicating treatment with immune-checkpoint inhibitors or other therapies targeting the immune system (7, 8).

**Figure 1:**
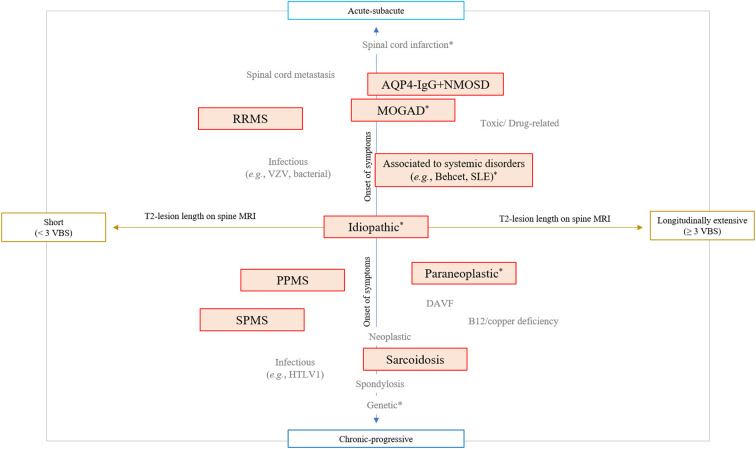
Integrated clinical-MRI approach for classification of myelopathies. The diagram schematically shows the most common myelopathy etiologies stratified based on the timing of symptoms presentation (acute vs. chronic progressive) and length of T2 lesion on spinal cord MRI (greater than 3 contiguous vertebral body segments or not). Immune-mediated myelopathies are highlighted in red boxes, while myelopathies that can present with a normal MRI are labeled with an asterisk (*). AQP4-IgG+NMOSD, aquaporin-4-IgG positive neuromyelitis optica spectrum disorder; DAVF, dural arteriovenous fistula; HTLV-1, human T-lymphotropic virus type 1; MOGAD, myelin oligodendrocyte glycoprotein-IgG associated disease; PPMS, primary progressive multiple sclerosis; RRMS, relapsing remitting multiple sclerosis; SLE, systemic erythematosus lupus; SPMS, secondary progressive multiple sclerosis; VBS, vertebral body segments; VZV, varicella zoster virus.

For this Research Topic, the characteristics of different immune-mediated myelopathies and common associated diagnostic tests have been extensively reviewed. Alkabie and Budrham reviewed the methodologies used for the testing of the two most common antibody-associated demyelinating myelitis, namely myelin oligodendrocyte glycoprotein antibody-associated disease (MOG-AD) and aquaporin-4 (AQP4) positive neuromyelitis optica spectrum disorders (NMOSD). They compare the performance of fixed and live cell-based assays, the yield of antibody detection in different specimens (serum and cerebrospinal fluid), and address the important topic of antibody titer cut-offs, which is particularly relevant for diagnostic accuracy of MOGAD. This latter point is also at the core of the original research of Manzano et al., who investigated the positive predictive value of serum anti-MOG antibody testing in a real-world institutional cohort, using well-established live cell-based assays. While on one hand the authors identify an assay cut-off 1:40 as the one yielding the highest positive predictive value, they also noted a sizeable number of cases with phenotype aligned with typical MOGAD that would have been missed with the use of such threshold, underscoring the need for further work in the optimization of diagnostic assays.

These articles altogether highlight how, even in the presence of useful biomarkers, the diagnosis of immune mediated myelopathies rests on clinical acumen, and the recognition of characteristic disease features remains the fundamental step to achieve diagnostic accuracy. To aid clinicians in the differential diagnosis of inflammatory myelopathies, Cacciaguerra et al. provide an overview of the key imaging features, pitfalls and mimics of the most common demyelinating syndromes and of some rare, recently characterized immune-mediated myelopathies. Recent years have seen an exponential rise in the number of biological agents that can be applied for the treatment of systemic inflammatory disorders and malignancies, and neurologists are increasingly challenged by possible secondary neurological manifestations of these treatments. This expanding field of iatrogenic myelopathies is the focus of the review of Gritsch et al., which provides useful recommendations for workup and management, while Passeri et al., provide an in-depth discussion of paraneoplastic myelopathies. Recognizing these rare but important causes of myelopathies has important treatment implications, and is now particularly relevant as the testing for many of the associated antibodies has become commercially available in the United States. Lastly, Fadda et al. compare the typical evolution, clinical and radiological short and long-term outcomes of spinal cord involvement in multiple sclerosis, MOGAD and AQP4 antibody positive NMOSD, emphasizing the importance of accurate diagnosis and prompt treatment initiation to alter the disease course of these disorders.

Our knowledge on many of the immune-mediated myelopathies discussed in these articles is still at its early stages, and many spinal cord syndromes observed in clinical practice continue to be labeled as idiopathic. While this field will likely continue to evolve in the upcoming years, physicians should familiarize with the current landscape of immune mediated myelopathies, as prompt recognition of these syndromes and early and targeted therapies are often key to provide the most optimal patient outcomes.

## Author contributions

GF, EF, and ES: study concept, design, and drafting the manuscript and figures. All authors contributed to the article and approved the submitted version.

## Conflict of interest

The authors declare that the research was conducted in the absence of any commercial or financial relationships that could be construed as a potential conflict of interest.

## Publisher's note

All claims expressed in this article are solely those of the authors and do not necessarily represent those of their affiliated organizations, or those of the publisher, the editors and the reviewers. Any product that may be evaluated in this article, or claim that may be made by its manufacturer, is not guaranteed or endorsed by the publisher.
